# Mapping value sensitive design onto AI for social good principles

**DOI:** 10.1007/s43681-021-00038-3

**Published:** 2021-02-01

**Authors:** Steven Umbrello, Ibo van de Poel

**Affiliations:** 1 Institute for Ethics and Emerging Technologies, University of Turin, Via Sant’Ottavio, 20, 10124 Turin, Italy; 2 Delft University of Technology, Faculty of Technology, Policy and Management, Jaffalaan 5, 2628 BX Delft, The Netherlands

**Keywords:** Value sensitive design, VSD, Artificial intelligence, AI4SG, Sustainable development goals, COVID-19

## Abstract

Value sensitive design (VSD) is an established method for integrating values into technical design. It has been applied to different technologies and, more recently, to artificial intelligence (AI). We argue that AI poses a number of challenges specific to VSD that require a somewhat modified VSD approach. Machine learning (ML), in particular, poses two challenges. First, humans may not understand how an AI system learns certain things. This requires paying attention to values such as transparency, explicability, and accountability. Second, ML may lead to AI systems adapting in ways that ‘disembody’ the values embedded in them. To address this, we propose a threefold modified VSD approach: (1) integrating a known set of VSD principles (AI4SG) as design norms from which more specific design requirements can be derived; (2) distinguishing between values that are promoted and respected by the design to ensure outcomes that not only do no harm but also contribute to good, and (3) extending the VSD process to encompass the whole life cycle of an AI technology to monitor unintended value consequences and redesign as needed. We illustrate our VSD for AI approach with an example use case of a SARS-CoV-2 contact tracing app.

## Introduction

There is ample discussion of the risks, benefits, and impacts of Artificial Intelligence (AI). Although the exact effects of AI on society are neither clear nor certain, AI is doubtlessly having a profound impact on overall human development and progress and it will continue to do so in the future [1–3]. AI is understood here as a class of technologies that are autonomous, interactive, adaptive, and capable of carrying out human-like tasks [4]. We are particularly interested in AI technologies based on Machine Learning (ML), which allows such technologies to learn on the basis of interaction with (and feedback from) the environment. We argue that the nature of these learning capabilities poses specific challenges for AI design. AI technologies are more likely than not to acquire features that were neither foreseen nor intended by their designers. These features, as well as the ways AI technologies are learning and evolving, maybe opaque to humans [5].

In this article, we build on and extend an approach to ethical design called value sensitive design (VSD). Although other tools for achieving responsible research and innovation have been proposed [6, 7], we specifically chose VSD as the design methodology due to its inherent self-reflexivity. VSD also emphasizes an engagement with both direct and indirect stakeholders as a fundamental part of the design process and the philosophical investigation of values [8, 9].

Past research has explored how VSD can be applied to specific technologies such as energy systems [10, 11], mobile phone usage [12], architecture projects [13], manufacturing [14, 15], and augmented reality systems [16], to name a few. Similarly, it has been proposed as a suitable design framework for technologies emerging in both the near- and long-term future. Examples include the exploratory application of VSD to nanopharmaceuticals [17], molecular manufacturing [18], intelligent agent systems [19], and less futuristic autonomous vehicles [20, 21]. Although these studies provide a useful theoretical basis for how VSD might be applied to specific technologies, they do not account for the unique ethical and technical issues presented by various AI systems.

To address these challenges, we suggest expanding VSD to include a set of AI-specific design principles. Here, we propose building on significant headway made recently in a number of AI for Social Good (AI4SG) projects that are becoming popular in various research circles [22]. Various AI-enabled technologies have already implemented practical, on-the-ground applications of the AI4SG principles [23]. This provides researchers with a solid foundation for the manifestation of ethics in practice. But AI4SG is nonetheless difficult and, given the multiplicity of research domains, practices, and design programs, its underlying principles remain fuzzy [24]. Still, some work has already been done to narrow down the essential AI4SG principles [3, 25].

This paper is organised as follows. Section 1 lays out the VSD framework. Section 2 explains why it is challenging to apply VSD to AI. Section 3 relates the motivations and description of the AI4SG principles as a way to address the specific challenges posed by AI to VSD. Section 4 outlines a design approach inspired by VSD and the AI4SG principles. To provide a preliminary illustration of this approach, Sect. 5 uses the example of a specific SARS-CoV-2 contact tracing smartphone application. Finally, Sect. 6 concludes the paper.

## Value sensitive design

Value sensitive design (VSD) is a principled approach to the design of new technologies that take values of ethical importance into account. The original approach was developed by Batya Friedman and colleagues from the University of Washington. As the approach grew more widespread, others developed it further (sometimes under somewhat different headings, such as ‘Values at Play’ or ‘Design for Values’ [26, 27]).

**Fig. 1:**
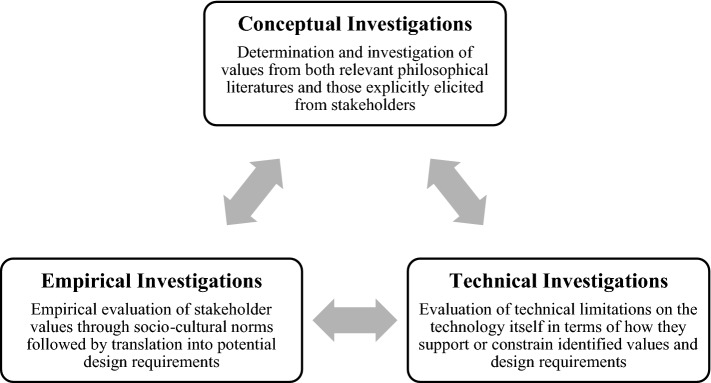
The recursive VSD tripartite framework was employed in this study. Source: [29]

At the core of the VSD approach is what Friedman et al. [28] call a tripartite methodology of empirical, conceptual, and technical investigations (see Fig. 1). Whether carried out consecutively, in parallel, or iteratively, these investigations involve: (1) empirical enquiries into relevant stakeholders, their values, and their value understandings and priorities; (2) conceptual enquiries into these values and their possible trade-offs; and (3) technical enquiries into value issues raised by current technology and the possibilities for value implementation into new designs.

One important issue in VSD is deciding how to identify which values should be taken into account through a concrete process [30]. Friedman et al. [28] propose a list of thirteen values that are important for the design of information systems (human welfare, ownership and property, privacy, freedom from bias, universal usability, trust, autonomy, informed consent, accountability, courtesy, identity, calmness, and environmental sustainability). Others have opposed a setlist of values, arguing that it is better to elicit values from stakeholders in a bottom-up approach [31, 32]. Both approaches probably have their advantages and disadvantages. A value list may well overlook values that are important in a specific situation, but absent from the list; a bottom-up elicitation may help uncover such values, but it is hardly watertight as stakeholders themselves may fail to articulate important values or crucial stakeholders may not have been identified. Stakeholder values, too, may not always have ethical importance that should be included in VSD.

When it comes to the identification of values in VSD processes for AI technologies, there are some important considerations. To begin, consensus about ethical issues specific to AI is now widespread. These issues are not raised by more conventional information and communication technologies, or at least not to the same degree [3]. The nature of AI thus carries two important implications for the issue of value identification. First, the original VSD list of values does not suffice for AI. Instead, one should consider values identified by an AI-specific entity as a starting point. For example, the EU high-level expert group on the ethics of AI lists the following values [33, 34]: respect for human autonomy, prevention of harm, fairness, and explicability. Second, some value list could be desirable to ensure that typical ethical concerns arising from AI are not overlooked. This is not to say that no other values should be included in the design of AI applications. They should, and some form of bottom-up elicitation may be relevant here.^1^ But any elicited list should be supplemented by principles to ensure that typical AI ethical issues are properly addressed. We propose recourse to the AI4SG meanings and principles, which the third section discusses in more detail.

## Challenges posed by AI

AI applications pose specific challenges for VSD that are largely due to their self-learning capabilities. This complicates the reliable integration of values into the design of technologies that employ AI. We start with a brief, imaginary illustration, then discuss the complications that AI raises for VSD in more general terms.

**Fig. 2:**
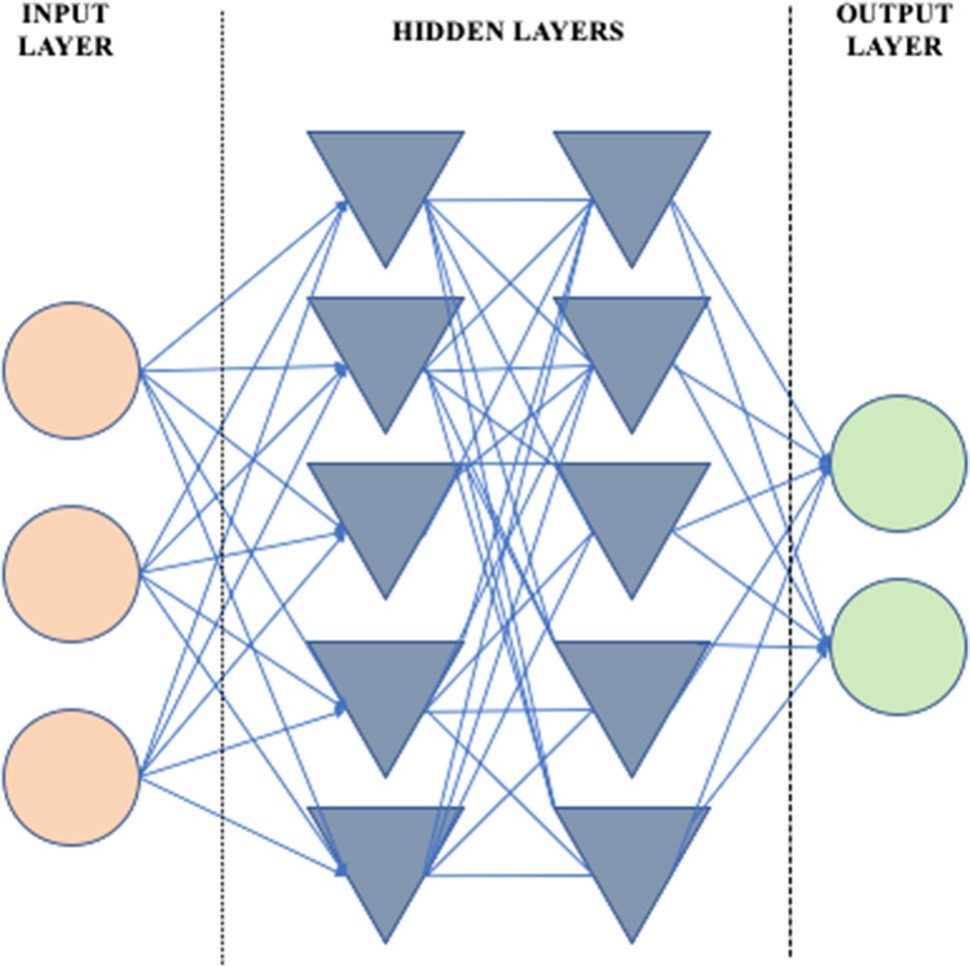
An artificial neural network

Suppose the tax department of a certain country wants to develop an algorithm that helps detect potential cases of fraud. More specifically, the application should help civil servants identify tax declarations that need extra or special scrutiny. Now, suppose the tax department chooses to build a self-learning artificial neural network for this task. An artificial neural network consists of a number of input units, hidden units, and one or more output units, as pictured in Fig. 2.

Let us suppose that the output unit or variable is simply a yes/no indicating whether a specific tax declaration needs additional scrutiny. The input variables (units) can be many. For example, they might include the amount of tax owed by a certain citizen, the use of specific tax exemptions, prior history (such as suspected fraud in the past), and any number of other personal details (age, sex, home address, etc.) (Fig. 2).

The units (variables) in the artificial neural network are connected as shown in Fig. 2. Connections between units can be weighted factors that are learned by the algorithm. This learning can be supervised or not [35]. If supervised learning is applied, the algorithm may learn to make calls on which tax declarations merit further scrutiny—calls that are similar to those of experienced civil servants within the tax department. In the case of unsupervised learning, information on which scrutinised cases led to the detection of actual fraud may be fed back into the algorithm. The algorithm may then be programmed to learn to select cases that have the highest probability of leading to actual fraud detection [36].

Now, one of the values that are obviously important in the design of such an algorithm is ‘freedom from bias’. This value was included on the original list proposed by Friedman and Kahn [37]. Friedman and Nissenbaum [38] define ‘bias’ in reference to “computer systems that systematically and unfairly discriminate against certain individuals or groups of individuals in favour of others” (p. 332). In traditional VSD, there are a number of ways this value may be implemented into algorithm design. First and foremost, it can be translated into design requirements that no variables within the artificial neural network (the nodes in Fig. 2) use as they may lead to an unwanted bias. Ethnicity, for example, maybe ruled out as a potential variable. But this is not enough to ensure the realisation of the value ‘freedom from bias’, as biases may also be introduced through proxy variables. Postal codes can be a proxy variable for ethnicity, so one may also need to rule out the use of such proxies to ensure ‘freedom from bias’ [39, 40].

Still, a self-learning algorithm can be biased due to the way it learns [41]. For instance, it could be biased because the training set for the algorithm is not representative or otherwise skewed. If a form of supervised learning is chosen, the algorithm might conceivably learn biases that exist in human judgments rendered for supervisory learning. Yet if these potential sources of bias are also excluded, there is still no guarantee that the resulting algorithm will be unbiased—and certainly not if a form of non-supervised (reinforcement) learning is chosen. One issue is that the resulting artificial neural network may be *described* as following a certain rule even if this rule was never encoded or (easily) derived from the nodes (variables) in the artificial neural network [c.f. 42]. In other words, the resulting algorithm might conceivably be described as following a rule that is somehow biased without this result being foreseeable or even clearly discernible.

Bias in the algorithm of this imaginary case could thus be *emergent* and *opaque*. Bias is emergent in the sense that it is an unintended and unforeseen consequence of the way the algorithm has learned. It is opaque in the sense that the bias may not be immediately clear or detectible from human inspection of the algorithm or artificial neural network.

The point generally applies beyond both this specific example and the value ‘freedom from bias’ (or fairness). Due to their self-learning capabilities, AI systems (especially those powered by ML) may develop features that were never intended or foreseen—or even foreseeable—by their designers.^2^ This means they may have unintended value consequences. It could even imply they might unintentionally ‘disembody’ values embedded in their original design [43, 44]. Unintended features may not always be discernible as they could be due to specific ways the algorithm has developed itself—ways that are hard or even impossible for humans to fully understand.

These issues are not necessarily insurmountable. In the case of the imaginary algorithm used by the tax office, technical solutions could at least make it much less likely for the system to develop in a biased direction: we might tell the algorithm to optimise itself not only in terms of effectiveness (as expressed in some number or rate of fraud detection), but also in terms of fairness (such as presenting a non-biased selection of cases for investigation) [41].

The salient point is that addressing emergence and opacity requires a set of design principles, or rather *norms*, that are not needed for traditional technologies. Some of these norms relate to technical or design requirements. Others concern organisation of the design process and the future life cycle of a product (continued monitoring, for instance). Still, others may address which AI techniques to use or not use. The next section looks at the AI4SG principles proposed as a way to address the specific challenges posed to VSD by AI.

## AI4SG meaning and factors

Thorough work on the harmonisation of AI4SG values was undertaken recently by Cowls et al. (2019) who focus on factors that are “particularly relevant” to AI (not exhausting the potential list of relevant factors). The seven principles that are particularly relevant for orienting AI design towards social good are: (1) falsifiability and incremental deployment; (2) safeguards against the manipulation of predictors; (3) receiver-contextualised intervention; (4) receiver-contextualised explanation and transparent purposes; (5) privacy protection and data subject consent; (6) situational fairness; and (7) human-friendly semanticisation [25].

**Fig. 3:**
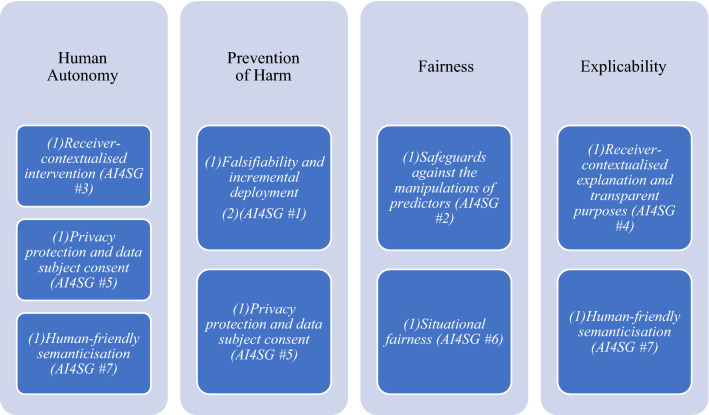
Relationship between higher-order values of the EU HLEG on AI and AI4SG norms

Although discussed separately, the seven factors naturally co-depend and co-vary with one another. Thus, they should not be understood as a rank-ordered hierarchy. Similarly, they each relate in some way to at least one of the four ethical principles laid out by the EU High-Level Expert Group on AI: *respect for human autonomy*, *prevention of harm*, *fairness*, and *explicability*. This mapping onto the more general values of ethical AI is not insignificant as any divergence from these more general values has potentially deleterious consequences. The function of the seven factors, then, is to specify these higher-order values into more specific norms and design requirements (Fig. 3).

We briefly describe the AI4SG principles below.

1. *Falsifiability and incremental deployment*
  Falsifiability is defined as “the specification…and the possibility of empirical testing” [25, p. 5]. This means other values implicated in AI design are predicated on their ability to be falsifiable or essential to the architecture of a technical system. Continued empirical testing must be undertaken in different contexts (which obviously cannot be exhausted without full deployment) to best ascertain the possible failures of a system. Hence there is a need for an incremental deployment cycle in which systems are introduced into real-world contexts only when a minimum level of safety warrants it.
2. *Safeguards against the manipulations of predictors*
  The manipulation of predictors can lead to a range of potentially deleterious outcomes for AI, moving away from the boons promised by the AI4SG values. This is described as the outcome of the “manipulation of input data as well as overreliance on non-causal indicators” [25, p. 57]. Along with the over-espoused but underthought value of transparency, overreliance on non-causal indicators often leads to the gamification of systems. Those who understand which inputs lead to which outputs can then gamify systems to yield their desired ends [5, 45].
3. *Receiver-contextualised intervention*
  The co-construction and co-variance of technology with the user implicates a delicate balancing act between how artefacts affect user autonomy. Within the context of technological design and development, user autonomy is a value of particular importance [46]. To balance the false positives and false negatives that can result in sub-optimal levels of user-based technology interventions, users can be given *optionality*. As one possible route for balancing interventions on autonomy, user optionality is contextualised from “information about users’ capacities, preferences and goals, and the circumstances in which the intervention will take effect” [25, p. 9].
4. *Receiver-contextualised explanation and transparent purposes*
  The aims of any given system must be transparent. That is, the operations carried out by a system should be explainable so as to be understood. The evermore ubiquitous deployment of AI systems is already underway. The need for explainability and transparency in their operations and goals has likewise garnered much attention due to the potential harms that can result from opaque goals and operations [5, 47, 48]. In relation to (3), information on system operations and objectives should be similarly receiver-contextualised [25].
5. *Privacy protection and data subject consent*
  Scholarship on privacy protection and subject consent is both rich and nuanced, encompassing decades of socio-ethical, legal, and other perspectives on the topics. As privacy forms the basis for good policy and just democratic regimes [49], AI4SG programs naturally make it an essential factor [50]. Tensions and boundaries between different levels or understandings of user data processing and use have already been explored, and there are nuanced proposals for how to adequately address them [51, 52]. Stakeholder data is foundational to the usability and efficacy of AI systems, so AI4SG systems seek a sufficient balance that respects the values of stakeholders in regards to data processing and storage.
6. *Situational fairness*
  As mentioned in (5), datasets are critical to the function of AI systems. Datasets can be biased on account of multiple factors (dataset collection, selection, categorisations, etc.). The resulting function of any given system will provide similarly biased results [5]. Biased decision-making can be of ethical importance because sets may involve ethically-relevant categories of data (such as race, gender, or age, among others) [38]. Recursive improvements to systems only exacerbate bias if designed/trained with biased datasets. The propagation of bias in datasets must thus be avoided if we are at attain AI4SG.
7. *Human-friendly semanticisation*

The task of managing or maximising the “semantic capital” of agents must be essential to the design of AI4SG systems. Floridi (2018) defines semantic capital as “any content that can enhance someone’s power to give meaning to and make sense of (semanticise) something” [53, p. 483]. AI allows for the automation of semanticisation, or making sense of things. If done haphazardly, the results may be ethically problematic. Arbitrary semanticisation can lead to results attributing meaning in ways that do not map onto our own understandings (random meaning-making). Dataset exposure may also be too limited, allowing for the propagation of narrow meanings; AI semanticisation will likewise be too narrow, thus limiting the redefinition or interpretation of things [54].

This section has condensed the seven essential factors critical to the design of AI4SG systems proposed by Floridi et al. (2020). Let us now see how these factors might help to overcome the challenges posed to VSD by AI, which we discussed in the previous section. We focus on the specific example given in Sect. 2. First, principle 6 would require “remov[ing] from relevant datasets variables and proxies that are irrelevant to an outcome” [25, p.18]. This is in line with the traditional VSD approach, but it is not enough as AI bias may be emergent and/or hidden (opaque). Principle 1 is particularly important for addressing the emergent character of bias, particularly the emphasis on incremental development. This is primarily a procedural requirement that requires monitoring and extending VSD to the full-life cycle of design, which we discuss in greater detail in the next section. To avoid opacity, AI4SG principles 4 and 7 are important. It should be noted that sometimes, the principles imply certain ML techniques should not be used.

## Adapting the VSD approach

To address the challenges posed to VSD by AI, we propose a modified VSD approach. The modifications we propose are threefold: (1) integrating AI4SG principles into VSD as design norms from which more specific design requirements can be derived; (2) distinguishing between values promoted by design and values respected by design to ensure the resulting outcome does not simply avoid harm but also contributes to doing good, and (3) extending the VSD process to encompass the whole life cycle of an AI technology to be able to monitor unintended value consequences and redesign the technology as needed. We begin by briefly explaining these new features and then sketch the overall process.

### Integrating AI4SG principles as design norms

**Fig. 4:**
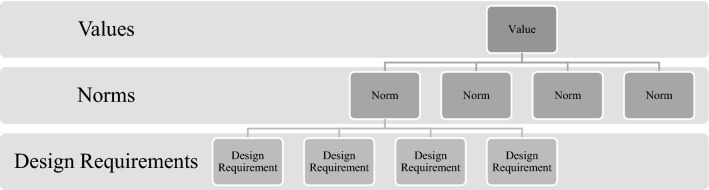
Values hierarchy. Source: Van de Poel, 2013

We propose mapping the AI4SG principles onto the ‘norms’ category used to translate values into technical design requirements, and vice versa, as outlined by Van de Poel (2013) (see Fig. 4).

### Distinguishing between promoted values and respected values

**Fig. 5:**
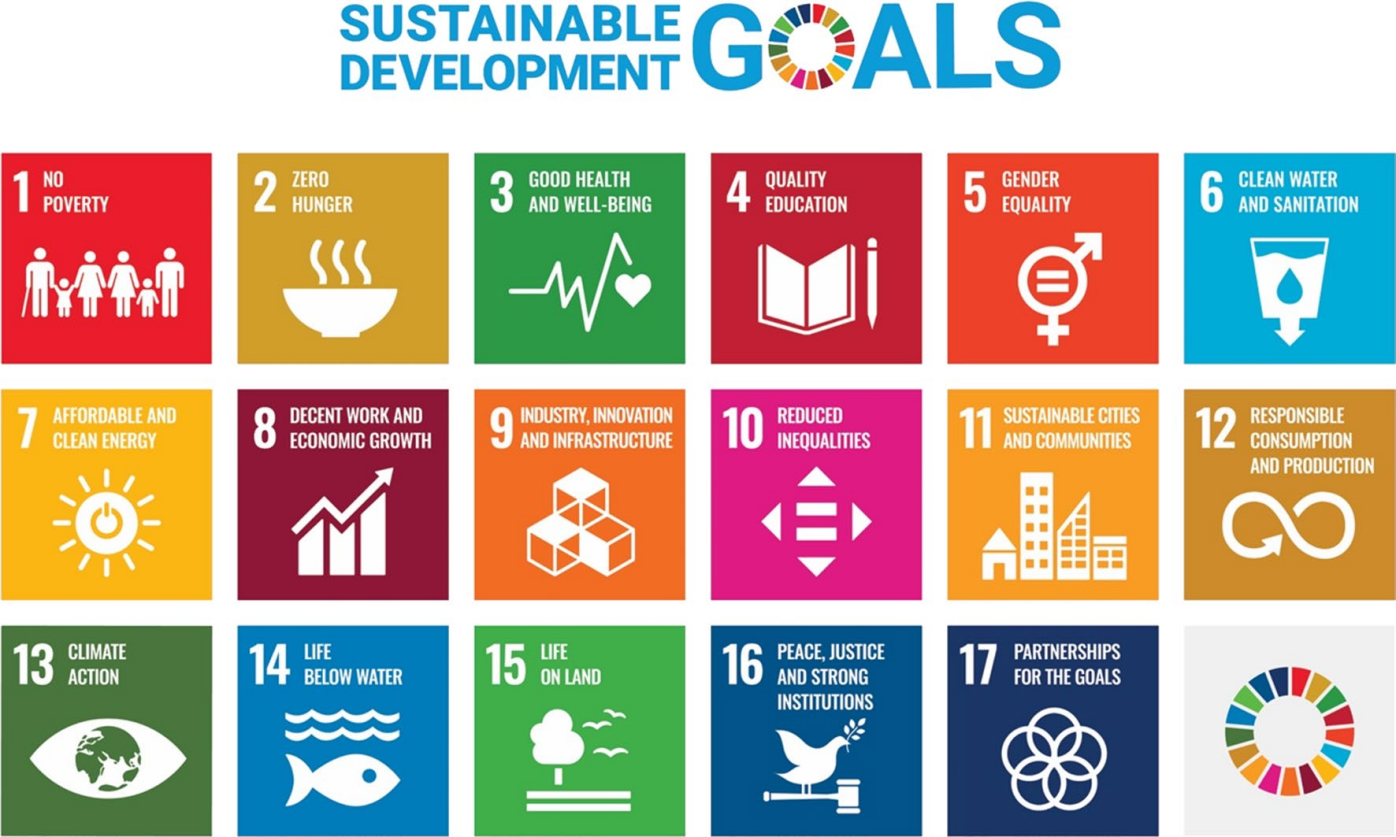
United Nations Sustainable Development Goals. Source: [55]

For a VSD approach to AI to be more than just avoiding harm and actually contributing to social good, there must be an explicit orientation toward socially desirable ends. Such an orientation is still missing in current proposals for AI4SG. We propose addressing this through an explicit orientation toward the Sustainable Development Goals (SDGs), proposed by the United Nations, as the best approximation of what we collectively believe to be valuable societal ends (Fig. 5).

### Extending VSD to the entire life cycle

To address the emergent and possibly undesirable properties that AI systems acquire as they learn, we propose extending VSD to the full life cycle of AI technologies. VSD will allow continued monitoring for potential unintended value consequences and technological redesign as needed [44, 56]. Indeed, the first AI4SG principle voices a similar idea: “AI4SG designers should identify falsifiable requirements and test them in incremental steps from the lab to the “outside world” [25, p. 7]. The need for ongoing monitoring arises from uncertainties accompanying the introduction of new technologies in society [57].

**Fig. 6:**
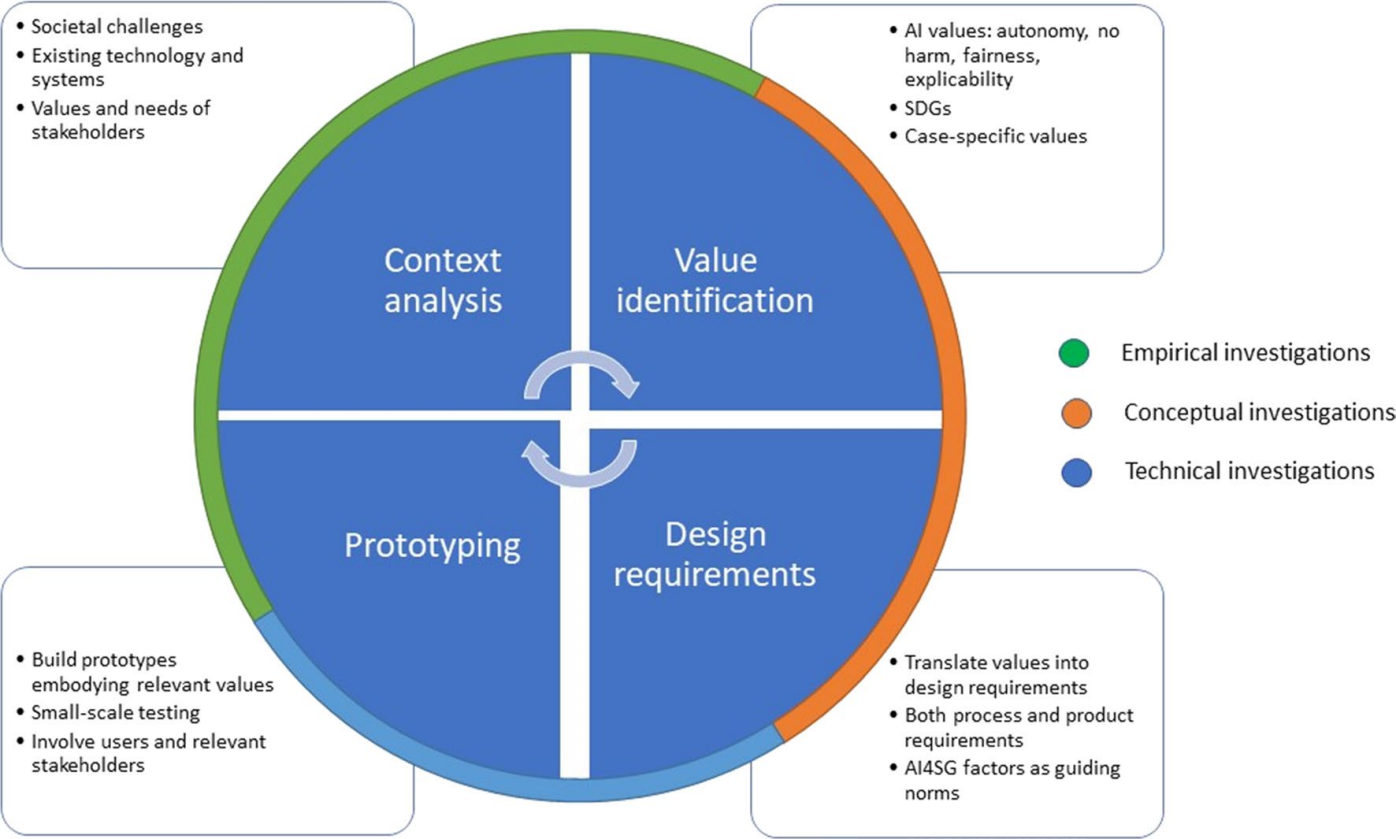
VSD design process for AI technologies

The resulting VSD process is illustrated in Fig. 6. Given that each AI system design has different uses and thus different value implications, the illustration serves as a general model for engineers to use as a guide throughout their design program.

We suggest that VSD for AI proceeds in four iterative phases briefly described below:

1. **Context analysis**: motivations for design differ across projects. For this reason, there is no normative starting point from which all designers begin. VSD acknowledges that technology design can begin with the discrete technology itself, the context for use, or a certain value as a starting point. In all cases, the analysis of context is crucial. Different contextual variables come into play to impact the way values are understood (in the second phase), both in conceptual terms as well as in practice, on account of different socio-cultural and political norms. The VSD approach sees eliciting stakeholders in sociocultural contexts as imperative. This will determine whether the explicated values of the project are faithful to those of the stakeholders, both directly and indirectly. Empirical investigations thus play a key role in determining potential boons and downfalls for any given context.
2. **Value identification**: the second phase concerns identification of a set of values to form a starting point for the design process. We suggest three main sources for such values. One source is the values promoted by the design, such as by deriving from the SDGs. Another source is those respected by the design, particularly the values identified in relation to AI (respect for human autonomy, prevention of harm or nonmaleficence, fairness and explicability [33, 58]). A final source is context-specific values that are not covered by the first two sources. Instead, they derive from analysis of a specific context in the first phase and of stakeholder values in particular. Moreover, it should be noted that phase two does not just involve empirical investigations. This phase has a distinct normative flavour in the sense that it results in the identification of values to be upheld in further design *from a normative point of view*. Phase two also involves conceptual investigations geared at interpreting (in context) and conceptualising relevant values.
3. **Formulating design requirements:** the third phase involves the formulation of design requirements on the basis of identified values (phase 2) and contextual analysis (phase 1). Here, tools such as the value hierarchy [59] can be useful for mutually relating values and design requirements, or for translating values into design requirements (Fig. 5). We suggest that the translation of values into design requirements is somewhat different from the sets of values formulated in the second phase. For example, the first set of values might be derived from the SDGs. These values will be promoted by the design. They are typically translated into design requirements formulated as criteria that should be met as much as possible. The second set of values are those that should be respected, particularly in regards to AI. Here, the AI4SG principles are especially helpful for formulating more specific design requirements. Requirements will most likely be formulated as constraints or boundary conditions rather than as criteria that should be achieved as much as possible. Boundary conditions thus set the deontological constraints that any design must meet to be ethically (minimally) acceptable. The third set of contextual values, the analysis of context and particularly stakeholders, will most likely play an important role in determining how values should be translated into design requirements.
4. **Prototyping**: the fourth phase is building prototype tests that meet design requirements. This idea is in line with what is more generally described in VSD as a *value-oriented mock-up, prototype, or field deployment* [8, p. 62]. We propose extending this phase to the entire life-cycle of AI technology. Even if technologies initially meet value-based design requirements, they may develop in unexpected ways and yield undesirable effects. They could fail to achieve the values for which they were intended, or they may have unforeseen side effects that require consideration of additional values [60]. In such cases, there is reason to redesign the technology and complete another iteration of the cycle.

To ensure adoptability and illustrate the efficacy of this approach, we provide timely examples below. These examples offer a clear illustration of how the process works by situating it within a figurative context for a specific AI system.

## The AI4SG-VSD design process in action: SARS-CoV-2 contact tracing apps

On Tuesday, April 7, 2020, the Robert Koch Institute (RKI)—the German federal research facility responsible for disease control and prevention – prompted German citizens with smartphones and smartwatches to voluntarily share their health data to track the spread of the COVID-19 virus [61]. The RKI is rolling out a new app called Corona Datenspende (Corona Data Donation), which allows users to voluntarily and anonymously share their health data. The data aids scientists in determining the symptoms correlated with COVID-19 infection as well as the distribution of infections across the nation. It further helps with gauging the efficacy of public health measures to ameliorate the situation. The app allows the user to record their age, height, weight, gender, health metrics (such as physical activity, body temperature, sleep behaviour, or heart rate), and postal code. Lothar Wieler, head of the RKI, said that the information collected by the app would “help to better estimate where and how fast Covid-19 is spreading in Germany” [62].

The RKI explicitly states that data collected from individual users is labelled with pseudonyms. Personal user information, such as names and addresses, remain private through the use of artificial identifiers to de-identify user data. However, this leaves open the possibility of re-identifying data subjects.^3^ Likewise, the machine learning systems underlying the app are designed to:

recognise symptoms that are associated with, among other things, a coronavirus infection. These include, for example, an increased resting heart rate and changes in sleep and activity behaviour. The donated data will only be used for scientific purposes. After careful preparation, the data flows into a map that visually shows the spread of potentially infected people down to the zip code level. [61]

Although the app is still in its infancy regarding stages of deployment, we can illustrate the design of the Corona Datenspende (albeit *ex post facto* in this case) using the framework described above (see Fig. 6).

### Context

In this case, the context for use—the COVID-19 pandemic—can be understood as the motivating factor behind a technological solution. The immediate (health) crisis demands swift action to stifle the further spread of the virus. Actions are also undertaken with the desire to return to less strict measures at some point post-pandemic. A *prima facie* analysis of the values at play here point to tensions between immediate concerns for *public health* and more enduring ones for *economic stability/prosperity.* Development of an app can be targeted to (attempt to) balance this tension, as a tracking and tracing app may assist in the resumption of certain societal activities such as travelling or work. By tracing potential infections, resumption can unfold in a way that still reduces health risks as much as possible.

### Value identification

#### Values promoted by the design: the UN sustainable development goals

The design of Corona Datenspende can be said to support the third SDG of “good health and well-being” (see Fig. 5). Although the impromptu technology was introduced in response to an immediate context, in situ deployment and use may encourage applications outside the original context (perhaps in other countries, or for other illnesses).^4^

#### Values respected by the design, with focus on those specific to AI: respect for human autonomy, prevention of harm (nonmaleficence), fairness, and explicability

*Respect for human autonomy*: here, autonomy refers to the balance between human decision-making power and its abdication to AI systems. Machines should be designed not only to promote human autonomy but also to constrain the abdication of too much decision-making power. This is especially true in areas where the value of human decision-making outweighs the efficacy of the machine’s decision-making capabilities [58]. Such respect aligns with the sixteenth SDG (Peace, Justice and Strong Institutions), particularly 16.7: *Ensure responsive, inclusive, participatory and representative decision-making at all levels* [55].

*Prevention of harm (nonmaleficence):* nonmaleficence means understanding systemic capabilities and limits to prevent potential risks and harms from manifesting in systems. When it comes to how individuals control their personal data, for example, questions of data privacy and security are often invoked [58]. RKI explicitly states that it does not collect personal user information beyond the level of postal codes (used to understand transmission densities). But privacy concerns exist at the community level nonetheless, particularly in regards to practices for storing, using, sharing, archiving, and destroying collected data. Risks of regional gerrymandering, targeted solicitation and/or discrimination are not excluded solely by delimiting data collection to the level of postal codes. Harm may also occur due to specific ways the app is used. This is especially true if the app is used to not only map the spread of the virus but also trace individuals as potential bearers of the disease and ‘risk factors’. We discuss these in more detail below, under contextual values.

*Fairness*: fairness is typically described and defined in different ways, creating ambiguity in meaning. It can also be specified across different points in the life cycle of AI technologies, including their relations with human beings. Here, fairness can be framed as justice. Floridi et al. (2018b) sum up various definitions of justice in three ways. The first definition of justice is using AI to correct past wrongs, such as by eliminating unfair discrimination. The second is ensuring the use of AI actually creates benefits that are shared (or at least shareable). The third is preventing the creation of *new* harms, such as undermining existing social structures. This general understanding of fairness as justice aligns directly with the sixteenth SDG (Peace, Justice and Strong Institutions).

*Explicability*: to support the other values, employed AI systems must be explicable. This means the inner workings of these systems must be *intelligible* (not opaque). There must also be at least one agent who is *accountable* for the way it works (an agent who understands the way the system works and is thus responsible for its actions [58]).

#### Context-specific values not covered by 1) and 2). These underpin analyses of specific contexts and focus especially on stakeholder values

Although the German government stated that Corona Datenspende would be voluntary, scholars pointed out that the app might nevertheless be applied in ways that endanger the voluntariness of use. For example, it could be used to allow access to certain services (such as public transport) or become required by employers for their employees. Such potential uses might, in turn, also invite individuals to misuse the app to retain maximum freedom of movement. Users might even conceal certain contacts by turning off their phones, which could again contribute to health risks.

Similar concerns were voiced in other countries. In the Netherlands, sixty scientists and experts wrote an open letter to the Dutch government warning about a number of risks and unintended consequences associated with a tracing and tracking app [63]. Among other things, they observed that such an app might lead to stigmatisation and discrimination. Depending on how it was used, it could endanger fundamental human rights such as the right of association. They also drew attention to the fact that an app might give a false sense of security, leading people to follow requirements for social distancing less strictly. This would ultimately increase, rather than decrease, health risks.

Many of the risks and potential side effects mentioned by scholars regarding SARS-CoV-2 apps map onto the values discussed above, particularly health values (under 1) and non-maleficence, and justice, autonomy, and explicability (under 2). For example, a false sense of security relates to the value of health. Privacy and voluntariness relate to the value of autonomy. Stigmatisation and discrimination relate to the value of fairness [e.g. 64–66]. Some values, such as the right of association or security against hacking and misuse, are less clearly related to one of the SDGs but could perhaps be subsumed under nonmaleficence.

More clearly, the issues show that we should consider values in context to gain full awareness of what is at stake and how we might translate our concerns into tangible design requirements. In this specific case, it is particularly important to consider the behavioural effects of contact-tracing apps. It is also crucial to view values within a broader systemic context. Even if a contextual value analysis fails to reveal completely new values, it will nonetheless be crucial for understanding which values are at stake for a specific application, how they should be understood in that specific case, and how they might translate into design requirements.

### Formulating design requirements

**Fig. 7:**
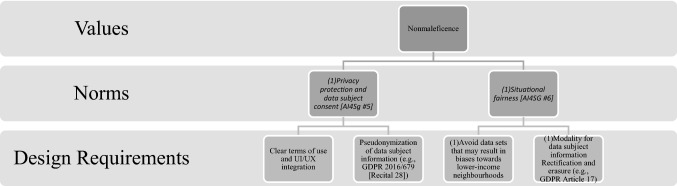
Translating the reduction of harm (nonmaleficence) into design requirements through AI4SG norms

To illustrate how tools such as the value hierarchy (Fig. 4) can be used to visualise and aid designers in translating abstract values into technical design requirements, we provide a specific instance of the tool below (Fig. 7). There are, of course, any number of iterations occupying any given vector in the hierarchy. This is just one example. Similarly, and to reiterate here, the desirability of using such a tool is the aid it provides designers. Designers are tasked with visualising and opening up potential design pathways. The tool helps in understanding the avenues for value translation, which are often abstract. These avenues translate values into concrete design requirements, and vice versa.

We chose the value of *nonmaleficence* because it is more abstract, thus illustrating the utility of the tool. Nonmaleficence was first translated through two of the AI4SG principles (5 and 6), and then into technical design requirements. In this paradigm, AI4SG principles are adopted as *norms* – rightly so, given their framing by Floridi et al. [67] as imperatives. Naturally, any given context for use, values, and technology specifics will implicate any number of combinations. There is no exclusive or exhaustive route that might satisfy value translation, which can move in a bottom-up direction (design requirements norms values) as well as the top-down diction (values norms design requirements) shown above (see also Longo et al. [67]). We could just as easily (and probably should) use *situational fairness* as the normative tool for operationalising other values, such as *explicability* (transparent dataset collection, use, storage, and destruction [for example, see 68]) and *justice* (promoting non-discriminatory laws and practices through unbiased compliance [for example, see the use of Fairness Warnings and/or Fair-MAML described by 69]).

At a functional level, the normative structure of AI4SG values supports avoiding (most) ethical harms associated with AI systems. However, it does not guarantee new AI applications will actively contribute to social good per se*.* Combined with *real* operationalisation of the SDGs, the higher level values listed above allow for the development of more salient AI systems that contribute to social good (or global beneficence). This multi-tiered approach, which couples AI-specific values with stakeholder values and their application to SDG attainment via AI4SG norms, can mitigate dangers posed by ethical white-washing. Such white-washing occurs when AI technologies that fail to respect some fundamental ethical principles are legitimised [70–72].

Regardless, this type of visualisation can be used across different sources for values. As listed above, sources include the SDGs and stakeholders. Visualisation helps determine how related values can produce technical design requirements that are both similar and different. Research projects could do this empirically, by taking any particular AI technology and providing thorough value-design requirement translations to determine the effectiveness of this approach. All in all, our aim is to help designers begin to design *for* various values more effectively. Often, these values are conflated erroneously or side-lined altogether.

### Prototyping

Prototyping involves building mock-ups of the technology in question according to design requirements laid out in the previous step. Technology is moved from the more controlled space of the lab or design environment and in situ. This, of course, implicates stakeholder values both directly and indirectly. Various design decisions may prove recalcitrant at this point. Alternatively, unforeseen recalcitrant behaviour could emerge to implicate other values. Assuming limited deployment of the technology, it could be recalled into the design space so corrective modifications might be implemented. Given the stakes at play and the urgency for amelioration, the crisis situation behind the inception of Corona Datenspende invited direct deployment rather than prototyping. But while tempting, this may ultimately prove an unwise course of action. AI systems possess significant risks, especially those that are predicated on such large quantities of data subjects. Small scale deployment or in-house testing of the efficacy and fidelity of the app’s underlying systems are a necessary (albeit insufficient) condition. Absent this condition, it is difficult to ensure the responsible development of an AI system of this type. Absent responsible development, the app is far less likely to achieve positive ethical/societal values (such as beneficence, justice, explicability, autonomy, and associated distal SDGs) or to reduce ethical AI risks (such as nonmaleficence).

It must be stressed that prototyping should not be limited to testing the proper technical function of the app. Prototyping must take into account behaviour, societal effects, and their subsequent impacts on values. Once again, the tracing and tracking app is a case in point. Some value issues, such as privacy, can be addressed through technical choices (e.g. pseudonymisation, local data storage, and the automatic destruction of data after a certain period of time). But other value concerns require insight into the behavioural impacts of this sort of app. Behavioural impacts are very hard, if not impossible, to predict reliably without some form of prototyping. At the very least, prediction requires small-scale testing in situ. It is therefore advisable to go through a number of trials for such apps, scaling up from very small-scale testing with mock-ups to test settings of increasing size (not unlike what is done in medical experiments with new drugs). Testing trajectories might also reveal new values brought to bear that need to be taken into account. Doing so would then trigger a new iteration of the development cycle.

## Conclusion

This paper discusses how AI systems can pose certain challenges to a value-sensitive design approach in technology. These result from the use of machine learning in AI, which creates two challenges for VSD. First, it may be not at all clear (to humans) how an AI system has learned certain things. The inherent opacity of AI systems requires paying attention to values such as transparency, explainability, and accountability. Second, AI systems may adapt themselves in ways that ‘disembody’ the values embedded in them by VSD designers. To deal with these challenges, we proposed an extension of VSD to the whole life cycle of AI systems design. More specifically, we have shown how the AI4SG principles iterated by Floridi et al. can be integrated as VSD norms when considering AI design. To integrate the AI4SG principles into a more systematic VSD approach, we proposed a design process consisting of four iterative basic steps: contextual analysis, value identification, translation of values into design requirements, and prototyping.

At the core of our model lies a two-tiered approach to ensuring values in the design of AI technologies. The first tier consists of a real commitment to contributing to social good (beneficence) through AI. The second tier involves the formulation of (and adherence to) a number of concrete AI4SG principles. AI4SG factors could help avoid most ethical harms, even without the first tier. But there is no guarantee at all that new AI applications will actively contribute to social good. So without the second tier, there is a danger of contributing to societal challenges. This occurs when SDGs are used to legitimise AI technologies that do not respect some fundamental ethical principles. Here lies the danger of ethical white-washing, which is already visible on the webpages of some large companies.

In addition to these two tiers of values, we have argued that contextual values are highly important (or at least the contextual interpretation of values from the two tiers). Contextual interpretation is necessary for understanding which values are at stake for a specific application, and how to translate relevant values into design requirements.

## Footnotes

1 Bottom-up approaches can be informed by the actual processes of participatory design and of responsible research and innovation, such as those by Abebe et al. 2020; Liao and Muller 2019; Whitman et al. 2018; Smith and Iversen 2018. They can also be informed by emerging regulation on constraining data collection practices as well as the design of AI systems (as relates to ‘protected characteristics’, human oversight, and informational roles, for example) [73, 74].

2 Although ML is chosen as the type of self-learning AI system in this paper, the VSD challenges posed by AI systems of this type are not exclusive to ML per se. In fact, most approaches to AI learning are combinatory/hybrid approaches involving different types of ML and ANN systems. Thus at the very least, they pose VSD challenges similar to the types explained here.

3 It bears mentioning that pseudonymisation is not equal to anonymisation. In fact, according to GDPR, only completely anonymised data are outside the scope of the regulation. Pseudonymised data does not have the same legal consequences as anonymised data due to a salient difference: anonymising data means to irreversibly delete any potential link to the person the data originally belonged to. Conversely, pseudonymising data means to de-identify data through artificial identifiers—leaving open the possibility of re-identifying data subjects. Although pseudonymisation is considered a secure means of storing personal information, it does not compare to anonymisation as the legal consequences are substantially different: the GDPR still applies to pseudonymised data [75, 76].

4 Goal 3 target 3.B more aptly aims to “support the research and development of vaccines and medicines for the communicable and noncommunicable diseases that primarily affect developing countries, provide access to affordable essential medicines and vaccines, in accordance with the Doha Declaration on the TRIPS Agreement and Public Health, which affirms the right of developing countries to use to the full the provisions in the Agreement on Trade Related Aspects of Intellectual Property Rights regarding flexibilities to protect public health, and, in particular, provide access to medicines for all” [55].
